# Audiological study of an elderly brazilian population

**DOI:** 10.1016/S1808-8694(15)30566-8

**Published:** 2015-10-19

**Authors:** Luís Cláudio do Carmo, José Alexandre Médicis da Silveira, Sílvio Antônio Monteiro Marone, Fabiana Gonçalez D’Ottaviano, Ludmila Lima Zagati, Eliane Maria Dias von Söhsten Lins

**Affiliations:** 1 PhD in Otolaryngology - FMUSP (University of São Paulo Medical School), Head of the ENT Residency Program - Hospital Santa Marcelina and Clínica Otorhinus.; 2 PhD in Otolaryngology - FMUSP (University of São Paulo Medical School), Head of the ENT Residency Program - Hospital Santa Marcelina and Clínica Otorhinus.; 3 PhD in Otolaryngology. Full Professor of ENT - PUC-Campinas and HCFMUSP.; 4 MSc. Student in ENT - FCMSCSP, Preceptor of the ENT Residency - Hospital Santa Marcelina and Clínica Otorhinus.; 5 MD. ENT Resident Physician - Hospital Santa Marcelina.; 6 MD. ENT Resident Physician - Hospital Santa Marcelina. Centro de Referência do Idoso (SES-SP).

**Keywords:** tonal audiometry, elderly, presbycusis

## Abstract

The Brazilian elderly population is growing, and already represents 8,6% of our total population. Environmental factors, lifestyle, gender and genetics impact the development of presbycusis, which reduces quality of life.

**Aim:**

investigate audiologic and vestibular complaints in the elderly; perform tonal audiometry and check to see if there are differences between genders.

**Study:**

Cross-sectional clinical prospective study.

**Materials and Methods:**

320 elderly patients (160 men and 160 women) were submitted to audiologic interview and tonal audiometry. The results were statistically analyzed by the following methods: ANOVA, Mann-Whitney and Chi-Squared.

**Results:**

audiologic and vestibular complaints (hearing loss, tinnitus, ear fullness, dizziness) were similar between the genders (except for dizziness: p<0,05); tonal audiometry showed a significant difference, with hearing loss in the high frequencies among men; and among women the curves were descending and flat. These results were statistically significant (P<0,001).

**Conclusion:**

our results lead us to conclude that, when the genders are compared, hearing loss in the elderly has similar symptoms; however, there are significant differences in tonal audiometry.

## INTRODUCTION

In Brazil, the elderly situation started to appeal to different areas of society after 1976, when the first seminars were held to discuss the problems of the elderly in our society. Looking at census data from IBGE^1^, One may see that in recent decades we have seen an accelerated growth of the Brazilian elderly population (individuals with 60 or more years of age).

The National Census of 1960 found that the Brazilian population was made up of 4.7% of elderly citizens, growing slowly to 5.1% in the 70's and reaching 6.1% of elderly citizens in the 80's^1^.

In the year 2000 National Census, the elderly population was reported to represent 8.6% of the country's population, which means a total of 14.5 million people. In relation to 1991, there was a growth of 35.5% in the total number of senior citizens. In that year the ratio of these people in the general population was of 7.3% or 10.7 million senior citizens^1^.

The increase in the elderly population may suggest an association with the increase in average life expectancy of the Brazilian population, social indication of better life quality and social well being. However, even if we have raised the average life expectancy, this does not mean that objective conditions have improved for the elderly^2^. Without any doubt, the sensorial privation accruing from hearing acuity represents one of the most terrible causes of isolation for the elderly^3^.

Berkowitz^4^ stated that approximately 55% of the American adults with enough hearing loss that interferes in their perception of speech are above 65 years of age. More recent statistics reveal that presbycusis affect approximately 25% of the American population between 65 and 74 years and 38% of the population above this age^5^.

Zwaardemaker^6^ was the first researcher to describe hearing loss in the higher frequencies and, later, the first to use the term presbycusis, which literally means “hearing loss in the elderly”, or, according to Davis (1962)^7^, the physiological hearing loss that happens with aging. Strictly speaking, presbycusis could be characterized as a “bilateral hearing loss for high frequency tones, caused by degenerative and physiological changes to the hearing apparatus with aging.”^8^.

Bunch^9^ showed the audiometric changes for high frequencies, documenting the drop in hearing function for frequencies above 500Hz, as age advances. He also observed that presbycusis affects the population in the following order: Caucasian men > Caucasian women > black men > black women.

Crowe et al.^10^ and Saxén^11^ were the first to associate inner ear pathologies with high frequency hearing loss. They described two pathological changes: one involving the organ of Corti and another involving the neurons of the spiral ganglion. The hearing loss indicated by the audiogram was associated to the organ of Corti atrophy, an atrophy of the cochlear nerve in the cochlea's basal portion.

Schuknecht^12^ classified presbycusis in four categories: sensorial, neural, metabolic and mechanical. Johnson & Hawkins^13^ created two more categories of presbycusis: vascular and central. And finally, Corso^8^ stressed that since these six types of presbycusis had been identified, they rarely occurred separately.

Kirikae et al.^14^ found degenerative changes in the auditory pathway nuclei and concluded that “senile changes in the nerve cells must be considered an important factor in the origin of presbycusis “.

Hansen & Reeske-Nilsen^15^ carried out a research in the cochlear nuclei of 12 brains from elderly people and concluded that there must be a reduction in the cochlear nucleus cells when compared to normal brains.

Suga & Linsay^16^ observed that the most prominent histopathology change in the inner ear was a reduction in the cell population of the spiral ganglion.

Schuknecht^17^ stated that with the advance of age, there is a reduction in the mitotic capacity of certain cells, reduction in nuclear protein, build up of pigments and other insoluble compounds in the cytoplasm and chemical alterations in the intercellular fluid.

In relation to predisposing factors for hearing loss in the elderly, Glorig & Nixon^18^ coined the term “sociocusys”, that is, the inevitable global effect of daily non-occupational exposure to noise, infections, drugs and acoustic trauma. Hearing loss with aging would be, for those authors, a combined effect of presbycusis, sociocusys and occupational noise.

Rosen et al.^19^ carried out an audiometric evaluation in men from a Sudanese Tribe, the Mabaans, who were not affected by noise, arteriosclerosis, smoking and drugs. Results from such study revealed a minimum hearing loss as age increased, as well as few indications of hearing loss associated with age or gender.

Weston^20^ obtained more important data comparing the life style of people living in urban and rural areas, and results showed that the hearing sensitiveness of people living in rural areas was significantly higher when compared to those living in cities.

Hinchcliffe^21^ studied a similar population in Jamaica and obtained results that showed very little difference in age-related hearing loss among Jamaican and Scottish senior citizens.

Gilad & Glorig^22^, have stated that age-related hearing loss would be the result of a cumulative effect of the various extrinsic factors added to genetically determined age models.

Anderson & Meyerhoff^23^ have also stated that the development of presbycusis seems to be also associated with other factors such as: diet, metabolism, cholesterol levels, blood pressure, arteriosclerosis, physical exercise, smoking, exposure to noise and stress.

According to Mader^24^, elderly people with sensorineural hearing loss would be better described as having hearing loss associated with aging, and such hearing loss encompasses: presbycusis or physiological hearing deterioration, sociocusys, including trauma by environmental noise, trauma by occupational noise, nosacusys (hearing loss component associated with other diseases or the effect of drug ingestion or medication use), genetic predisposition.

According to Gates^25^, audiometric studies carried out in isolated, non-industrialized populations identified a minimum hearing loss in function of age, differently from industrialized populations, reinforcing that social conditions such as noise exposure, alcohol ingestion and smoking, nutritional characteristics and genetic factors can worsen hearing loss in the elderly.

The study carried out by Cruickshanks et al.^26^ revealed that smokers have almost a two-fold hearing loss when compared to non-smokers. Nonetheless, the association between smoking and dyslipidemias seem to further worsen the development of presbycusis, as well as a prior history of isolated blood hypertension, or associated with other risk factors which may worsen the clinical picture^27^.

The interaction between noise exposure and presbycusis was also investigated and showed that there are more complex mechanisms than a simple summation of losses. In experiments with animals, they were exposed to low-to-moderate noise intensity for a long period of time, in short and high intensity duration this mechanism does not seem to be exact^28^.

About the presence of symptoms associated with hearing loss in the elderly, Bora et al.^29^ stated that presbycusis, unbalance, vertigo and tinnitus represent the most common otolaryngological complaints of geriatric patients.

According to Schneider et al.^30^, tinnitus is one of the most important symptoms in otoneurology, right after vertigo, nausea and hearing loss, and all these complaints tend to worsen with age. Ahmad & Seidman^31^ stated that the prevalence of tinnitus increases with age and that there is greater incidence when the hearing loss in the elderly is noise-induced.

Rosenthall & Karlsson^32^ state that tinnitus would be constantly present in 8 to 15% of the cases, usually when there are higher hearing losses and, as an intermittent symptom, in about 20 to 42%. In the study carried out by Nondahl et al.^33^, prevalence varied between 5.7 and 8.2% of the elderly patients. Now, Sindhusake et al.^34^ studied 2015 elderly and they found a prevalence of 30.3%, and 48% of them had bilateral complaints, and only 6% received some treatment for this problem.

On the other hand, labyrinth symptoms may also accompany the symptoms of hearing loss and would accrue from the incidence of degenerative alterations in the vestibular system, such as degeneration of the saccular nerve bundle and of its neuroepithelium, as well as its saccular otolithic system and, in a lesser degree, that of the utricular otolithic system^35^. Katsarkas^36^ believes that 12.22% of the patients admitted to the vertigo ward of the Royal Victoria Hospital were 70 years old or more on their first medical visit.

According to Huang et al.^37^, in elderly patients, vertigo and balance disorders would be caused by different types of central and peripheral vestibular pathological dysfunctions. By the same token, a functional reduction in the vestibular organs and system affected by the pathological processes of aging and other concomitant diseases, as well as environmental and psychogenic factors may also be involved^37^.

The goals of the present investigation were to study the hearing and labyrinthine complaints from a sample of the Brazilian elderly population and characterize the audiometric profile of these elderly, assessing the differences between genders.

## MATERIALS AND METHODS

We carried out a cross-sectional study in a secondary level outpatient ward, in a region where most of the elderly population is needy (classes D and E)^1^. The research project was submitted and approved by the Ethics in Research Committee (ERC) of the institution (CAAE 010.0.253.000-06).

The series comprehended a group of 320 individuals with one or more audiological or associated labyrinthine complaints (hearing loss, tinnitus, ear fullness, dizziness), with a minimum age of 60 years (“elderly) for males and females.

The subjects were asked about the presence of the aforementioned complaints. We also investigated the presence of worsening factors of hearing loss in the elderly, such as systemic blood pressure, diabetes mellitus, hyperlipidemia, smoking and exposure to occupational noise, in order to better characterize the population under study.

We performed threshold tonal audiometry, determining the thresholds for both air as bone conduction. Hearing loss type was defined according to Santos & Russo^38^, in: normal, conductive, mixed and sensorineural. We also used the terms descending, ascending, horizontal and irregular in order to subdivide the hearing loss according to this same reference. Classification of the degree of hearing loss according to the threshold, as established by Kemker^39^: <25dBHL normal; 26-40dBHL mild; 41-55 dBHL moderate; 56-70 dBHL moderately severe; 71-90dBHL severe; 91-110 dBHL profound. In order to characterize frequencies we used Davis's scale^7^, or averages of 500, 1000 and 2000Hz (low) and 3000 and 4000Hz (high).

In order to compare the results between genders, we used the ANOVA and Mann-Whitney tests to analyze the quantitative variables and the Chi-Squared for the qualitative variables. The significance level (α) to reject the null hypothesis was fixed at 5% (that is, p < 0.05). We used the EpiInfo, version 3.3.2 software package.

## RESULTS

The analysis of the elderly (n=320), according to age and gender, presented two homogeneous groups, of normal distribution and with a statistically significant difference (Table 1).

**Table 1:** Distribution of the elderly individuals (n=320), according to age in years and gender.

GENDER	N	MEAN	SD	MIN	MEDIAN	MAX	VC	p*
MALE	160	71,70	7,03	60	71	95	0,09	
FEMALE	160	69,81	6,82	60	69	95	0,09	<0,05

N: number of individuals; SD: standard deviation; MIN: minimum age; MAX: maximum age; VC: variation coeficient; p:p-value

* evaluation by the ANOVA and Mann-Whitney tests

**Graph 1:**
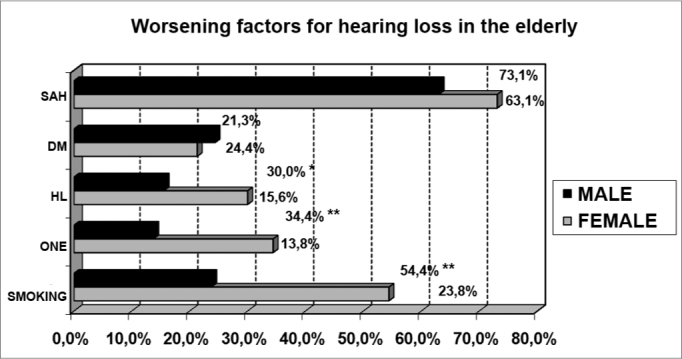
Elderly distribution (n=320) according to the presence of worsening factors and gender. - HAS: high blood pressure; DM: diabetes mellitus; HL: hyperlipidemia; ERO: exposure to occupational noise; FUMO: smoking; Chi-squared: *p<0.001

The investigation of the factors that worsen the hearing loss in the elderly showed a high percentage of cardiovascular and metabolic disorders, and also extrinsic factors, such as smoking and noise exposure (Graph 1). There was an association between the presence of some of these factors and gender (hyperlipidemia: p<0.05; smoking and noise exposure: p<0.001).

**Graph 2:**
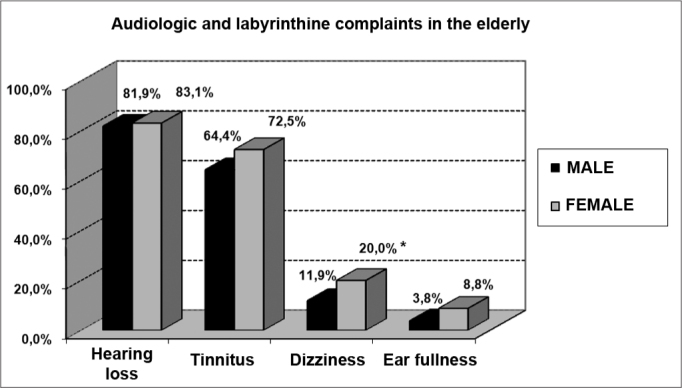
Elderly distribution (n=320) according to the presence of audiological or labyrinthine complaints and gender. - Chi-squared: *p<0.05

**Graph 3:**
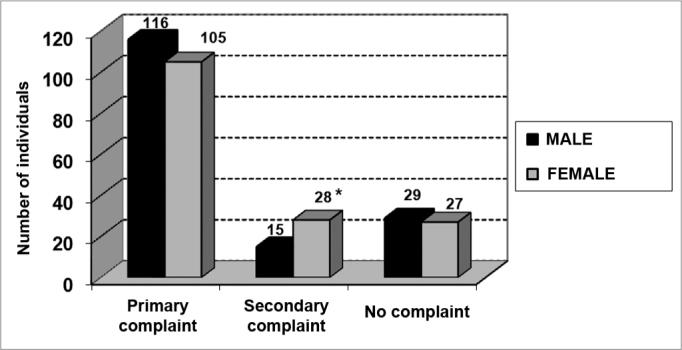
Elderly distribution (n=320) according to hearing loss and gender. - Chi-squared: *p<0.05

Among the symptoms investigated, hearing loss was the most frequent, followed by tinnitus, dizziness and ear fullness (Graphs 2 and 3). We found a relationship between gender and dizziness only (p<0.05).

**Graph 4:**
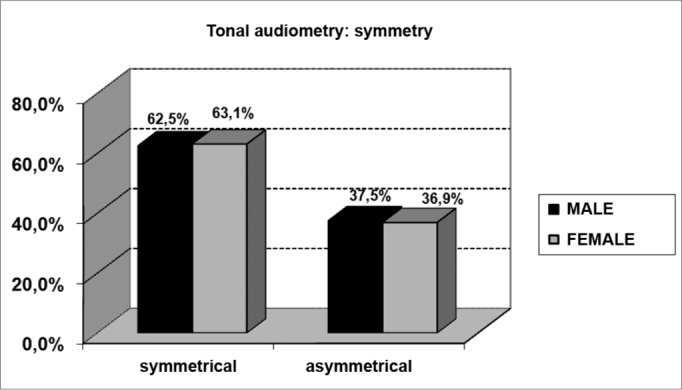
Elderly distribution (n=320) according to ears symmetry in tonal audiometry and gender. - Chi-squared: p>0.05

**Graph 5:**
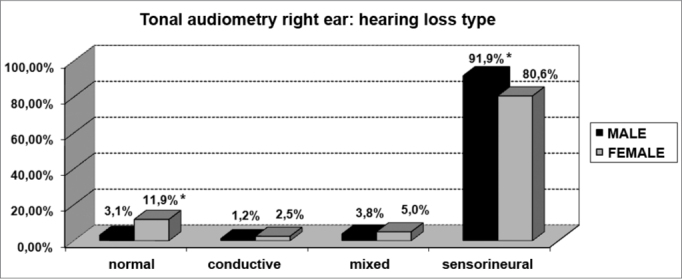
Elderly distribution (n=320) according to the hearing loss in the right ear tonal audiometry and gender. - Chi-squared: *p<0.05

**Graph 6:**
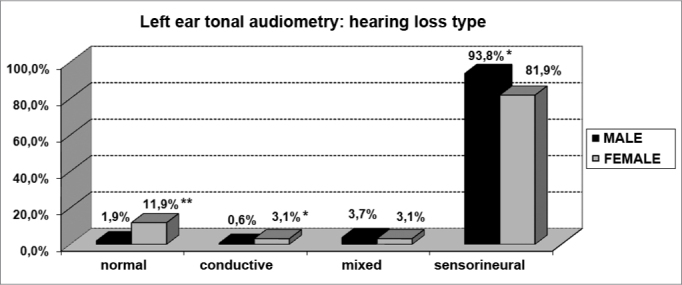
Elderly distribution (n=320) according to the hearing loss in the left ear tonal audiometry and gender. - Chi-squared: *p <0.001

As far as tonal audiometry is concerned, we noticed an important ratio of asymmetry between the ears, without association with gender (Graph 4). Sensorineural losses were the ones most commonly found, and there were significant differences between genders (Graphs 5 and 6).

**Graph 7:**
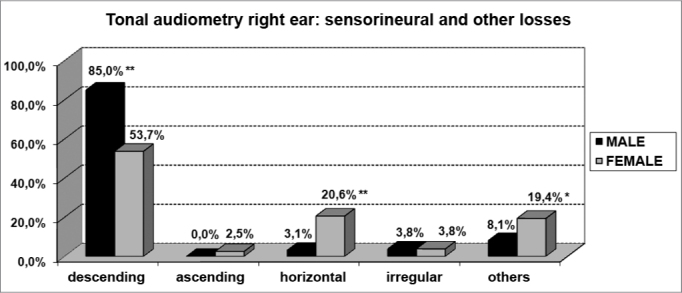
Elderly distribution (n=320) according to the type of sensorineural hearing loss in the right ear tonal audiometry and gender. - Chi-squared: *p<0.001

**Graph 8:**
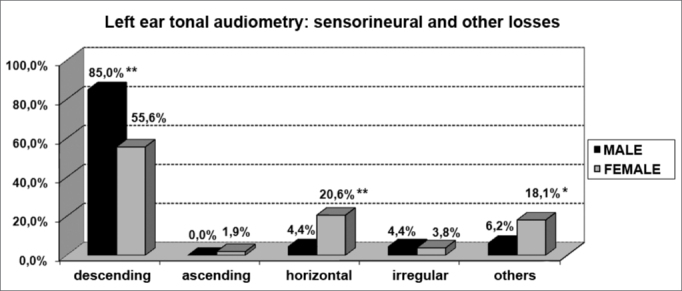
Elderly distribution (n=320) according to the type of sensorineural hearing loss in the left ear tonal audiometry and gender. - Chi-squared: *p<0.001

About the most common type of curve, there was a predominance of deeper hearing loss in the higher frequencies when compared to the lower frequencies (descending curves) for both genders (Graphs 7 and 8). Nonetheless, for females the presence of horizontal curves was very frequent, showing a relationship between both variables (p<0.001).

With the results of the tonal thresholds and considering a moderately severe hearing loss (>55dBHL), in low and high tones, as a sign of hearing loss worsening, we found a relationship between aging and a hearing loss worsening only in men (p<0.05). When we considered a moderate level of hearing loss (>41dB NA), such association becomes even more evident, with p<0.001 (Table 2).

**Table 2:** Distribution of the elderly individuals (n=320), according to age in years and the presence of moderate to moderately-severe hearing loss in tonal audiometry.

HEARING LOSS	N	MEAN	SD	MIN	MEDIAN	MAX	VC	p*
M RE MS								
NO	114	70,51	6,42	60	69	88	0,09	
YES	46	74,63	7,66	61	75,5	95	0,10	<0,05
M LE MS								
NO	117	70,63	6,50	60	69	88	0,09	
YES	43	74,60	7,64	61	75	95	0,10	<0,05
M RE Md								
NO	61	69,08	5,82	60	68	86	0,08	
YES	99	73,31	7,24	60	74	95	0,10	<0,001
M LE Md								
NO	55	68,85	5,69	60	68	86	0,08	
YES	105	73,19	7,22	60	73	95	0,10	<0,001
F RE MS								
NO	130	69,35	6,56	60	69	95	0,09	
YES	30	71,76	7,66	61	69,5	85	0,11	>0,05
F LE MS								
NO	127	69,45	6,56	60	69	95	0,09	
YES	33	71,27	7,70	61	68	85	0,11	>0,05
F RE Md								
NO	75	68,80	6,72	60	68	95	0,10	
YES	85	70,69	6,83	60	70	86	0,10	>0,05
F LE Md								
NO	76	68,22	6,61	60	67	95	0,10	
YES	84	71,24	6,73	60	70	86	0,09	<0,05

N:number of individuals; SD:standard deviation; MIN:minimum age; MAX:maximum age; VC:variation coefficient; p:p-value M:male; F:female; RE: right ear; LE:left ear; MS:moderately severe loss; Md: moderate loss.

* evaluation by the ANOVA and Mann-Whitney tests.

## DISCUSSION

Based on the results obtained from the descriptive analysis of the elderly population investigated, we can notice that the two groups formed (males and females), as far as age is concerned, have normal distribution, with similar data variability, thus favoring statistical inference reliability.

We observed a high incidence of hearing loss worsening factors in the population studied, especially those with high blood pressure (63.1%, men; 73.1%, women), smoking and noise exposure in men (54.4% and 34.4, respectively) and hyperlipidemia in women (30.0%). Many authors, from Rosen et al. and his research with the Mabbans until more recent ones such as Huang, have investigated the interaction of cardiovascular diseases and other extrinsic factors with presbycusis^18^, ^19^, ^20^, ^21^, ^22^, ^23^, ^24^, ^25^, ^26^, ^27^, ^28^, ^37^. The data from our study illustrates the need to control these disorders in order to properly manage elderly patients with labyrinthine and auditory complaints.

The incidence of tinnitus (64.4% and 72.5%, for men and women, respectively) in our study was higher when compared to other studies involving senior citizens (Rosenthall 20 to 42%; Nondahl, 5.7 to 8.2%; Sindhusake 30.3%)^32^, ^33^, ^34^. Such difference may be associated with the fact that we included in our study only elderly with one or more hearing complaints. Thus, we would have a greater percentage of tinnitus associated with deeper hearing losses^32^.

About dizziness, there was a relationship with gender (p<0.05), being more common in the elderly women (20.0%). In their investigation, Kamierczak & Doroszewska concluded that despite not finding any difference in the distribution of dizziness between elderly men and women, vertigo was more frequent in women. Moreover, multisensory deficits, drugs or systemic diseases common to senior citizens may cause vertigo. ^40^.

According to the results from tonal audiometry, we noticed an increase in auditory thresholds, especially in higher frequencies in both genders. However, a worse hearing loss in higher frequencies was more evident in men when compared to women, and this may be seen through the higher percentage of descending curves among males (85.0% in both ears) when compared to women (53.7% and 55.6%, for right and left, respectively).

On the other hand, we observed a significant relationship between females and audiometric high and low tone alterations. This relationship was characterized by the presence of horizontal curves very frequent in elderly women (20.6% in both ears). And this was not seen among men (3.1% and 4.4%, right and left, respectively).

The stepwise rise in hearing thresholds with frequency increase was already expected in both genders, since presbycusis is a sensorineural hearing loss that develops first and more severely in the higher frequencies, because, according to the histology studies carried out by Crowe et al. and Schuknecht, there is an organ of Corti dystrophy and that of the cochlear branch of the vestibule-cochlear nerve in the cochlear base^10^, ^12^.

Many other authors have stated that in the elderly there is a higher rise of auditory thresholds in the higher frequencies^3^, ^8^, ^9^, ^18^, ^21^, ^41^, ^42^. For gates, this variation would not be different according to gender, even if men have poorer thresholds^25^.

Nonetheless, there would be some differences in hearing loss development when we compare men to women. Males usually present more pronounced hearing loss in the higher frequencies; while in women it happens in the lower frequencies, at around 500Hz^3^, ^25^. Goetzinger et al., assessing hearing in elderly between 60 and 98 years, reached the same conclusions^43^.

Studies carried out in our settings have also confirmed a gradual hearing loss as the person ages, and it is more pronounced in the higher frequencies when compared to the lower frequencies for both genders. However, women have more horizontal audiometric curves than men^41^.

In his study with 912 individuals of both genders, with ages varying between 18 and 65 years, Corso concluded that women had better tonal thresholds for higher frequencies than men, even though the opposite happens for the lower frequencies (250 and 500Hz)^8^.

The author explained this phenomenon stating that:

1. age-related hearing loss onset is more stepwise for women than for men;
2. once started, the hearing loss develops faster for women, although the relative hearing loss in low frequencies for them are greater than those in men;
3. age-related hearing loss rate is more uniform in women; for men this rate varies with age in a less uniform fashion.

Moller and Kryter related this fact to women having less hearing loss for the higher frequencies because of their work as housewives, less exposure to noxious noises than men. Thus, their hearing thresholds reflect, more homogeneously, the hearing loss accruing from the aging process^44^, ^45^.

And finally, when we tried to correlate the age increase with hearing loss worsening, we found a worsening in the tonal thresholds only in men (Table 2). In women, there was no worsening in audiometric thresholds in older subjects, contrary to what was found by Corso, Moller, Kryter^8^, ^44^, ^45^.

## CONCLUSIONS

The results found in this study led us to conclude that, when we compare genders, hearing loss in the elderly has -except for dizziness - similar symptoms and significant differences in tonal audiometry.
